# Role of introvert and extrovert personalities in perception of COVID-19’s impact, psychological state, knowledge, infection, and preparedness preferences

**DOI:** 10.1186/s12889-025-22293-3

**Published:** 2025-03-31

**Authors:** Jiaying Li, Daniel Yee Tak Fong, Mandy Man Ho, Edmond Pui Hang Choi, Kris Yuet Wan Lok, Jung Jae Lee, WenJie Duan, Janet Yuen Ha Wong, Chia-Chin Lin

**Affiliations:** 1 School of Nursing, Li Ka Shing Faculty of Medicine, University of Hong Kong, Hong Kong SAR, China; 2 Johns Hopkins University, Baltimore, MD USA; 3 Department of Social Work, East China University of Science and Technology, Shanghai, China; 4 Hong Kong Metropolitan University, Hong Kong SAR, China; 5 School of Nursing, The University of Hong Kong 5/F, Academic Building 3 Sassoon Road, Pokfulam, Hong Kong

**Keywords:** Introverts, Extroverts, Response to COVID-19, Psychological response, Knowledge, Preparation preference

## Abstract

**Background:**

The role of introversion and extraversion in shaping pandemic responses remains understudied in the field of public health. This study aimed to comprehensively investigate differences in perceptions of COVID-19’s impact, psychological status, knowledge of COVID-19, infection rate, and preferred preparations among introverts and extroverts.

**Methods:**

This study utilized a cross-sectional design. From May to June 2022, an online survey was conducted, involving 1,990 adults in Hong Kong. Regression analyses were employed to identify personality differences across 58 outcomes of interest. To account for multiplicity, adjustments were made using the Holm-Bonferroni method.

**Results:**

Extroverts reported a greater increase in having a meal at home (*adjusted p [adj.p]* < 0.001), while introverts’ sleep quality decreased more (*adj.p* < 0.001). Although no statistical difference was detected between the decrease they showed in emotional stress (*adj.p* = 1.000) and mental burden (*adj.p* = 1.000), introverts had higher levels of anxiety (*adj.p* = 0.006), depression (*adj.p* < 0.001), and fear (*adj.p* = 0.026), whereas extroverts had stronger out of control feelings (*adj.p* = 0.010). Besides, extroverts had higher self-rated knowledge on COVID-19 knowledge (*adj.p* = 0.016) and prevention (*adj.p* < 0.001). Moreover, extroverts perceived higher importance in online consultation with doctors, instant personalized health by online chatbot, online courses, instant streaming courses, medicine delivery, online shopping, and food delivery (all *adj.p* < 0.05).

**Conclusions:**

Introverts could benefit most from interventions addressing sleep quality, anxiety, depression, fear, and knowledge promotion about COVID-19, while extroverts could benefit most from approaches that address feeling out of control. Extroverts had higher preferences for online consultations, instant personalized health via online chatbots, streaming courses, online courses, and medicine delivery, emphasizing the importance of considering personality in field of telemedicine, e-health, and remote medicine practice. These findings have important implications for pandemic response and preparedness, highlighting the role of personality in public health emergencies.

## Introduction

The COVID-19 pandemic has precipitated profound global changes in both physical and social environments by affecting individuals' physical, psychological, financial, and social well-being [1]. Extroverted and introverted individuals respond to environment differently [2]. Extroverts, naturally inclined towards external activities and social engagements, often seek stimulating experiences to compensate for their low cortical arousal levels, a trait Eysenck associates with higher emotional intelligence [3]. Conversely, introverts, characterized by a preference for internal reflection and a generally reserved demeanor, navigate their higher cortical arousal levels by limiting social interactions [3]. These personality-based predispositions imply the existence of different responses to the disruptions caused by the pandemic.

Research indicates that extroverts, with their pronounced preference for social interactions, might exhibit reluctance toward adhering to physical distancing and stay-at-home guidelines [4], possibly showing a propensity for higher vaccine hesitancy rates [5]. However, these findings are contentious, with another study highlighting no substantial evidence supporting the link between extraversion and vaccine refusal [6]. Furthermore, extroverts tend to adopt adaptive coping mechanisms, notably by seeking socio-emotional support [7]. While existing studies have mostly focused on the effectiveness of public health measures, they often overlook how the pandemic has altered extroverts' lifestyles and health outcomes, including their preferences for future pandemic preparedness. Additionally, there is a notable gap in research concerning personality differences in psychological states, COVID-19 pandemic-related knowledge, and infection rates, which necessitates further investigation.

Regarding the changes brought from the pandemic on lifestyle and health outcomes as well as psychological states, while the existing research has primarily centered on the pandemic's mental health impacts, revealing higher anxiety and depression among introverts [8–11], and with limited evidence showing that extroverts had more unhealthy eating and excessive screen time during the pandemic [12], a significant gap remains in understanding the full spectrum of lifestyle changes and their health implications. Broadening our understanding from mental health to a comprehensive overview of lifestyle and health impacts, covering social engagement, sleep quality, physical activity, and more, can inform personalized public health approaches tailored to personality traits to enhance well-being and lower post-pandemic health risks.

Additionally, understanding the relationship between personality traits and COVID-19 knowledge, infection status, and e-health literacy is crucial. Initial findings suggest extroverts might spread rumors and underestimate the importance of social distancing, emphasizing the need to customize containment strategies to personality differences [4, 13]. Yet, a detailed comparison of how different personalities influence COVID-19 knowledge, e-health literacy, and infection rates is lacking. Investigating these relationships to develop targeted measures that improve education, raise awareness, and lower infection rates across diverse personality profiles is necessary.

Moreover, while the significance of pandemic preparedness across various systems like healthcare and food supply has been extensively discussed, the individual preference for preparedness, particularly through the lens of personality traits, has been overlooked [1]. Our research fills this critical void by evaluating and contrasting these personal preferences, offering novel insights into how personality-specific strategies could inform future pandemic policies. This approach not only sheds light on uncharted facets of pandemic response but also equips policymakers with the knowledge to devise more effective, personalized public health interventions.

Aiming to bridge the abovementioned research gaps, this study examines the differences in perceptions of COVID-19's impact, psychological status, knowledge of COVID-19, infection rates, and preferred preparations among introverts and extroverts. This focused investigation not only enriches our theoretical understanding but also offers practical guidance. It equips stakeholders to navigate current and future health crises more effectively by informing about personality-driven vulnerabilities, highlighting domains that demand targeted interventions, and suggesting communication strategies and preparedness plans.

## Method

### Study aims and design

This cross-sectional study aimed to comprehensively investigate differences in perceptions of COVID-19’s impact, psychological status, knowledge of COVID-19, infection rate, and preferred preparations among introverts and extroverts.

### Setting

This study conducted online surveys to engage the Hong Kong community amid the pandemic. The study was conducted in a period that followed approximately two months after the Omicron surge (February 28 to March 6, 2022). During this time, social distancing measures were actively enforced. The average daily confirmed cases were 544 in a population of 7.41 million, according to government data [14].

### Participants and sample size

The eligible participants were Chinese reading adults in Hong Kong, ages 18 years and older. A total of 1900 completed questionnaires were received. For our current purpose of examining the differences between extroverts and introverts in 32 outcomes, we accounted for the issue of multiple comparisons by using the conservative Bonferroni adjustment to control the overall false positive error rate at 5%. That is, we set the nominal level of significance for each comparison as 0.05/32 = 0.00156. To achieve 80% power to detect a small effect size *f*^2^ of 0.02 using linear regressions with 4 predictors in each, we need 1,105 responses [15]. Therefore, the sample size of 1,900 was adequate for our purposes.

### Variables and measurements

#### Socio-demographics

Sociodemographic characteristics included gender, age, marital status, education, employment, perceived social rank, weight, height, body mass index (BMI), whether they were health professionals, whether they had children under the age of 18 and how many, and the number of people they lived with.

### Personality

We assessed participants' personality types using a single self-reported item: "Do you identify more as an introvert or an extrovert?" This approach aligns with methodologies employed in previous studies [11, 16]. Respondents were provided with two options: A) Introverted ("I prefer solitary activities or spending time with one or two close friends and tend to be quiet and reserved") and B) Extroverted ("I am sociable, enjoy lively gatherings, and like meeting new people").

#### Changes in lifestyles and health since the COVID-19 pandemic

The development, translation, and validation of lifestyle and health outcome items was outlined in previous published protocol [17]. The development of the questionnaire began with a comprehensive review of existing literature, complemented by discussions among a diverse team of experts in public health, nursing, and nutrition based in Hong Kong. This collaborative effort resulted in an initial English version of the questionnaire, crafted to align with the research goals and maintain face validity. To address cultural relevance, consultation with country-specific experts guided the adaptation of the questionnaire items, which were then translated into the necessary local languages through a systematic forward–backward translation method. A pilot test involving a minimum of ten participants for the Chinese version was implemented to ensure the clarity of the questionnaire items across different countries and to maintain data uniformity.

Participants were asked to self-report changes in their 18 lifestyles and 14 health outcomes due to COVID-19 compared to before the pandemic, using a 5-point Likert scale from 1 to 5 (1 = Substantially less to 5 = Substantially more, and 3 indicated no change). The lifestyle areas examined included food types in daily meals, consumption of fruits and vegetables, consumption of frozen food/food products, consumption of snacks, soft drinks/juices/other sugary drinks, having a meal at home, cooking at home, eating takeout food, taking alternative medicine or natural health products, taking oral supplements/vitamins, smoking tobacco, alcohol consumption, duration of sitting, duration of screen time, frequency of exercise, duration of exercise, type of exercise, and overall amount of exercise. Health outcomes included weight, appetite, physical health, sleep quality, quality of life, mental burden, emotional distress, family disputes, social support provided, social support received, social activities, income, working hours, and economic burden.

### Psychological states during the COVID-19 pandemic

#### Anxiety and depression

Anxiety and depression were assessed using the PHQ-4 scale, validated for use among Hong Kong residents with excellent composite reliability (Ω = 0.80–0.86) [18]. This instrument measures four mental distress symptoms experienced by respondents in the past two weeks, including an item such as "Feeling nervous, anxious, or on edge." Responses are captured on a 4-point Likert scale, ranging from 0 = "not at all" to 3 = "nearly every day," where higher scores denote more severe symptoms of anxiety or depression. In this study, the Cronbach’s alpha values for the anxiety and depression subscales, as well as the overall scale, were 0.790, 0.823, and 0.876, respectively.

#### Fear

Fear was measured using a validated Chinese version of the Fear of COVID-19 Scale, demonstrating high reliability with a Cronbach’s alpha of 0.93 [19]. An example item is “The thought of COVID-19 scares me,” with responses scored on a 5-point Likert scale ranging from 1 (strongly disagree) to 5 (strongly agree). Higher aggregate scores indicate a greater degree of fear. In this study, the scale's Cronbach’s alpha was 0.940.

#### Out of control

The 7-item Out-of-Control Scale, validated among Chinese adults with a Cronbach’s alpha of 0.91, was used to assess perceived control loss during the COVID-19 pandemic. An item example is, “I cannot handle daily problems and troubles.”Responses were on a 6-point Likert scale from "Strongly Disagree" to "Strongly Agree," where higher scores indicated more perceived loss of control [20]. The scale's Cronbach’s alpha in this study was 0.851.

### COVID-19-related knowledge, e-health literacy, and infection status

#### COVID-19-related knowledge

Six items from the World Health Organization (WHO) behavioral survey on COVID-19 were used to assess participants' knowledge and susceptibility, three of which were about perceived knowledge, and three were about perceived severity [21]. An example, “How would you rate your knowledge level on COVID-19?”, used a 7-point Likert scale from "Very Poor" to "Very Good." Higher scores indicated better knowledge.

#### E-health literacy

The Chinese version of the 8-item eHEALS Scale, with a Cronbach's alpha of 0.834, evaluated electronic information-seeking behavior related to COVID-19 [22]. An example item, "I know how to find helpful health resources on the Internet," used a 5-point Likert scale from "Strongly Disagree" to "Strongly Agree." Higher scores indicated greater health literacy. The scale’s Cronbach’s alpha in this study was 0,949.

#### COVID-19 infection status

Two additional items from the WHO survey on COVID-19 status, including whether participants themselves or someone in their immediate social circle had become infected, were used to assess their and their social circle infection status [21].

#### Preference for 13 aspects of pandemic preparations

Participants were asked to rate, on a 5-point Likert scale from 1 to 5 (1 = Not important to 5 = Extremely important), their perceived importance of a list of items on possible preparation during a pandemic. These items included online consultations with doctors (e.g., Zoom, Skype), instant personalized health advice by online chatbot, telephone health advice, online courses, instant streaming courses (e.g., Zoom, Skype), receiving health information through email, receiving health information through text (e.g., SMS, WhatsApp), receiving health information from social media (e.g., Facebook, Instagram, Twitter), receiving health information from mobile app, get medicine prescribed in a hospital visit and follow-up in a community pharmacy, medicine delivery, online shopping, and food delivery. Previously published protocols reported the development of the questionnaire regarding possible preparations [17].

### Data collection

Data were collected from May 2 to June 24, 2022. Participants were randomly selected from a panel list of individuals who had previously participated in other territory-wide studies and were invited to participate through email or text messages. Interested participants followed a provided link to self-complete the questionnaire.

### Data analysis

The response data collected online were exported into Excel files. All statistical analyses were performed by R 4.1.1 (v4.1.1; R Core Team, 2021). Descriptive statistics were used to summarize the socio-demographics. Continuous variables conforming to the normal distribution were summarized by means and standard deviations, whereas categorical variables were described by frequency, utilizing the package 'dplyr'. Age, education level, and perceived social rank were assumed to be confounders [23–25]. Linear or logistic regression was conducted for each outcome by personality, adjusting for age, education level, and perceived social rank, utilizing the package 'stats'. To control for the family-wise error rate and counteract the problem of multiple comparisons, the Holm–Bonferroni method was used to adjust *p*-values, utilizing the package 'stats'. The adequacy of all regression models was assessed by examining the standardized residuals. Statistical significance was set at* p* < 0.05.

## Results

### Respondents’ socio-demographics

During May 2-June 24, 2022, a total of 2500 emails or text messages were sent. Of which, 102 email addresses or phone numbers were invalid. Of the remaining 2398 contacted individuals, 1900 (79%) completed our survey. There were no missing data in the responses. Among them, 1019 (53.6%) were female and 1019 (53.6%) were extrovert. Participants ranged in age from 18–24 to > 65 years old. Table 1 summarizes and compares respondents’ socio-demographics by personality.

**Table 1:** Summary and comparisons of respondents' socio-demographics by personality type (*n* = 1900)

Socio-demographics	Mean (SD) / n (%)	
	**Introvert (*n* = 881)**	**Extrovert (*n* = 1019)**
Age group (1–10)^a^	5.25 (2.88)	4.90 (2.63)
Height (m)	1.64 (0.08)	1.65 (0.08)
Weight (kg)	63.22 (9.00)	64.32 (10.13)
BMI (kg/m^2^)	23.40 (2.52)	23.62 (2.90)
Number of children less than 18 years old	0.38 (0.70)	0.42 (0.72)
Number of people lived with	3.33 (1.14)	3.37 (1.14)
Marital status		
Married/Cohabitation/Common-law	539 (61.2%)	623 (61.1%)
Separated/Divorced/Widowed	37 (4.2%)	34 (3.3%)
Single	305 (34.6%)	362 (35.5%)
Gender		
Female	519 (58.9%)	557 (54.7%)
Male	362 (41.1%)	462 (45.3%)
Highest level of education attained		
Primary or below	90 (10.2%)	56 (5.5%)
Secondary	348 (39.5%)	443 (43.5%)
Associate degree	78 (8.9%)	102 (10.0%)
College	118 (13.4%)	137 (13.4%)
Bachelor	228 (25.9%)	267 (26.2%)
Graduate	19 (2.2%)	14 (1.4%)
Occupation (multiple choice)		
Job seeking	41 (4.7%)	45 (4.4%)
Laid off	13 (1.5%)	7 (0.7%)
Not in workforce	95 (10.8%)	118 (11.6%)
Retired	78 (8.9%)	50 (4.9%)
Self-employed	28 (3.2%)	34 (3.3%)
Student	52 (5.9%)	42 (4.1%)
Working (> = 40 h/wk)	441 (50.1%)	553 (54.3%)
Working (1–39 h/wk)	133 (15.1%)	170 (16.7%)
Practicing health professional		
No	864 (98.1%)	980 (96.2%)
Yes	17 (1.9%)	39 (3.8%)
Having children less than 18 years of age		
No	649 (73.7%)	717 (70.4%)
Yes	232 (26.3%)	302 (29.6%)
Perceived social rank, 1 = lowest to 5 = highest	2.82 (0.78)	2.97 (0.77)

^a^The age groups were defined as follows: 1 to 10 corresponded to the age ranges of 18–24, 25–29, 30–34, 35–39, 40–44, 45–49, 50–54, 55–59, 60–64, and 65 years and older, respectively

### Personality differences in perception of COVID-19’s impact on lifestyle and health changes

Table 2 assesses the influences of personality on COVID-19’s impact with adjustment of age, education, and perceived social rank. After accounting for multiplicity, extroverts had significantly more increase in having a meal at home (*adjusted p* < 0.001) and better maintained sleep quality (*adjusted p* < 0.001) than introverts. Personality showed no association with the other 30 perceived COVID-19’s impact (*adjusted p* > 0.05).

**Table 2:** The influences of personality on perceptions of COVID-19’s impact on lifestyle and health changes (*n* = 1900)

COVID-19’s impact	Descriptions: mean (SD)		Regression: extrovert vs introvert^a^	
	**Introvert (*n* = 881)**	**Extrovert (*n* = 1019)**	**b (95% CI)**	Adjusted p**
Lifestyles impact (1 = Substantially less to 5 = Substantially more)				
1. Having a meal at home	3.85 (0.86)	4.01 (0.83)	0.16 (0.09, 0.24)	< 0.001
2. Cooking at home	3.82 (0.86)	3.93 (0.81)	0.12 (0.04, 0.19)	0.060
3. Duration of sitting	3.65 (0.81)	3.75 (0.74)	0.11 (0.04, 0.18)	0.060
4. Consumption of fruits and vegetables	3.28 (0.71)	3.37 (0.72)	0.09 (0.02, 0.15)	0.216
5. Frequency of exercise	2.44 (0.93)	2.58 (0.91)	0.11 (0.02, 0.19)	0.300
6. Duration of exercise	2.43 (0.93)	2.56 (0.92)	0.10 (0.02, 0.19)	0.336
7. Smoking tobacco	2.83 (0.76)	2.91 (0.66)	0.07 (0.01, 0.14)	0.609
8. Eating takeout food	3.42 (0.96)	3.54 (0.92)	0.09 (0.01, 0.18)	0.620
9. Consumption of frozen food/food products	3.61 (0.78)	3.65 (0.77)	0.05 (−0.02, 0.12)	1.000
10. Consumption of snacks	3.10 (0.88)	3.15 (0.85)	0.02 (−0.06, 0.10)	1.000
11. Soft drinks/juices/other sugary drinks	3.14 (0.84)	3.18 (0.82)	0.02 (−0.06, 0.09)	1.000
12. Food types in daily meals	3.04 (0.68)	3.04 (0.69)	−0.02 (−0.08, 0.05)	1.000
13. Taking TCM or natural health products	3.02 (0.58)	3.05 (0.53)	0.04 (−0.01, 0.09)	1.000
14. Taking oral supplements/vitamins	3.27 (0.66)	3.29 (0.65)	0.02 (−0.03, 0.08)	1.000
15. Alcohol consumption	2.80 (0.77)	2.77 (0.77)	−0.04 (−0.11, 0.03)	1.000
16. Duration of screen time	3.69 (0.83)	3.75 (0.78)	0.05 (−0.02, 0.12)	1.000
17. Type of exercise	2.43 (0.94)	2.53 (0.88)	0.08 (0.00, 0.10)	1.000
18. Overall amount of exercise	2.43 (0.93)	2.52 (0.91)	0.07 (−0.02, 0.15)	1.000
Health-related impact (1 = Substantially less to 5 = Substantially more)				
1. Sleep quality	2.88 (0.73)	3.03 (0.70)	0.12 (0.06, 0.19)	< 0.001
2. Physical health	2.84 (0.77)	2.97 (0.69)	0.09 (0.03, 0.16)	0.140
3. Weight	3.36 (0.71)	3.44 (0.68)	0.09 (0.02, 0.15)	0.216
4. Appetite	3.05 (0.67)	3.13 (0.61)	0.07 (0.01, 0.13)	0.336
5. Family disputes	3.28 (0.65)	3.20 (0.60)	−0.07 (−0.13, −0.01)	0.336
6. Income	2.61 (0.78)	2.54 (0.80)	−0.07 (−0.14, 0.00)	0.893
7. Quality of life	2.76 (0.75)	2.79 (0.78)	0.00 (−0.07, 0.07)	1.000
8. Mental burden	3.48 (0.73)	3.45 (0.67)	−0.01 (−0.07, 0.05)	1.000
9. Emotional distress	3.47 (0.72)	3.40 (0.66)	−0.05 (−0.12, 0.01)	1.000
10. Social support provided	3.08 (0.58)	3.04 (0.56)	−0.04 (−0.10, 0.01)	1.000
11. Social support received	3.18 (0.63)	3.15 (0.61)	−0.01 (−0.06, 0.05)	1.000
12. Social activities	2.14 (0.98)	2.15 (1.04)	−0.02 (−0.11, 0.07)	1.000
13. Working hours	2.81 (0.83)	2.75 (0.82)	−0.06 (−0.13, 0.02)	1.000
14. Economic burden	3.37 (0.80)	3.37 (0.81)	0.03 (−0.05, 0.10)	1.000

Personality types (introverts vs. extroverts) were considered as independent variables, with each variable on a separate line as a dependent variable. After completing all regressions, the Holm-Bonferroni method was used to adjust the *p*-values from the regressions

^a^Adjusted for age, education, and perceived social rank

^**^Adjusted *p* values were *p*-values calculated using Holm–Bonferroni method

### Personality differences in psychological status during the COVID-19 pandemic

Table 3 summarizes the effects of personality on respondents’ psychological status during the COVID-19 pandemic, adjusting with age, education, and perceived social rank. Extroverts had a stronger feeling of out of control than introverts (*adjusted p* = 0.010). However, introverts had a higher level of fear (*adjusted p* = 0.026), anxiety (*adjusted p* = 0.016), and depression (*adjusted p* < 0.001) than extroverts.

**Table 3:** The influences of personality on psychological status (*n* = 1900)

Psychological status (score range)	Descriptions: mean (SD)		Regression: extrovert vs introvert^a^	
	**Introvert (*n* = 881)**	**Extrovert (*n* = 1019)**	***b* (95% CI)**	Adjusted p**
Fear (8–40)	21.65 (7.23)	20.71 (7.29)	−0.75 (−1.40, −0.09)	0.026
Out of control (7–42)	33.74 (8.02)	34.91 (7.83)	0.79 (0.24, 1.34)	0.010
Anxiety (2–8)	3.20 (1.33)	2.96 (1.13)	−0.17 (−0.28,−0.06)	0.006
Depression (2–8)	3.04 (1.38)	2.75 (1.12)	−0.24 (−0.35,−0.13)	< 0.001

Personality types (introverts vs. extroverts) were considered as independent variables, with each variable on a separate line as a dependent variable. After completing all regressions, the Holm-Bonferroni method was used to adjust the *p*-values from the regressions

^a^Adjusted for age, education, and perceived social rank

^**^Adjusted *p* values were *p*-values were calculated using Holm–Bonferroni method

### Personality differences in COVID-19-related knowledge, infection status, and e-health literacy

The influences of personality on COVID-19-related knowledge, infection status and e-health literacy are shown in Table 4. After accounting multiplicity, extroverts only showed significantly higher self-rated COVID-19’s knowledge (adjusted *p* = 0.016), and prevention of the spread of COVID-19 (adjusted *p* < 0.001).

**Table 4:** The influences of personality on COVID-19 related knowledge, e-health literacy, and infection status (*n* = 1900)

Variables (score range)	Descriptions: mean (SD) / n (%)		Regression: extrovert vs introvert^a^	
	**Introverts (*n* = 881)**	**Extroverts (*n* = 1019)**	***b/OR (*95% CI)**	Adjusted p**
How would you rate your knowledge level on COVID-19? (1–7)	4.26 (0.93)	4.43 (0.92)	0.13 (0.05, 0.21)	0.016
How would you rate your knowledge level on how to prevent spread of COVID-19? (1–7)	4.30 (0.90)	4.50 (0.95)	0.16 (0.08, 0.25)	< 0.001
Do you think you have adequate knowledge about COVID-19? (1–7)	4.30 (0.96)	4.44 (0.97)	0.10 (0.01, 0.18)	0.144
During the pandemic, how susceptible do you consider yourself to an infection with COVID-19? (1–7)	4.16 (1.25)	4.27 (1.32)	0.12 (0.01, 0.24)	0.185
During the pandemic, how severe would contracting COVID-19 be for you? (1–7)	4.33 (1.23)	4.18 (1.29)	−0.15 (−0.26, −0.03)	0.091
During the pandemic, how severe is the spread COVID-19 in your community? (1–7)	4.43 (1.28)	4.52 (1.25)	0.10 (−0.02, 0.21)	0.368
E-health literacy (8–40)	26.90 (6.54)	27.64 (6.40)	0.30 (−0.23, 0.83)	0.540
Whether you have been infected with the COVID-19 (Yes/No)	278/603	325/694	0.96 (0.79, 1.17)	0.702
Whether someone in your immediate social circle has been infected COVID-19 (Yes/No)	568/313	694/325	0.85 (0.70, 1.03)	0.368

Personality types (introverts vs. extroverts) were considered independent variables, with each variable on each line taken as the dependent variable. After completing all the regressions, the Holm-Bonferroni method was used to adjust the *p*-values from the regressions

^a^Adjusted for age, education, and perceived social rank

^**^Adjusted *p* values were *p*-values calculated using the Holm–Bonferroni method

### Personality differences in preparations preference

Table 5 presents the influences of personality on the perceived importance of possible preparations for the pandemic. After adjusting the *p* values for multiplicity, extroverts had a significantly higher perceived importance in online consultation with doctors (*adjusted p* = 0.002), instant personalized health by online chatbot (*adjusted p* = 0.006), online courses (*adjusted p* = 0.002), instant streaming courses (*adjusted p* = 0.005), medicine delivery (*adjusted p* = 0.043), online shopping (*adjusted p* = 0.002), and food delivery (*adjusted p* = 0.002).

**Table 5:** The influences of personality on the perceived importance of preparations for a pandemic (*n* = 1900)

**Preparations** (1 = Not important to 5 = Extremely important)	**Descriptions: mean (SD)**		**Regression: extrovert vs introvert**^a^	
	**Introverts (*n* = 881)**	**Extroverts (*n* = 1019)**	***b (*95% CI)**	Adjusted p**
Online consultation with doctors	2.87 (1.08)	3.08 (1.11)	0.19 (0.09, 0.29)	0.002
Online shopping	3.49 (1.12)	3.72 (1.08)	0.18 (0.08, 0.27)	0.002
Food delivery	3.66 (1.08)	3.84 (1.03)	0.18 ( 0.08, 0.27)	0.002
Online courses	2.68 (1.05)	2.90 (1.11)	0.19 (0.09, 0.28)	0.002
Instant streaming courses	2.80 (1.10)	3.02 (1.16)	0.18 (0.08, 0.28)	0.005
Instant personalized health by online chatbot	2.64 (1.12)	2.84 (1.16)	0.18 (0.07, 0.28)	0.006
Medicine delivery	3.87 (1.11)	4.00 (1.00)	0.13 (0.04, 0.23)	0.043
Telephone health advice	2.98 (1.08)	3.10 (1.17)	0.11 (0.01, 0.21)	0.229
Get medicine prescribed in a hospital visit/follow-up in a community pharmacy	3.64 (1.10)	3.72 (1.02)	0.10 (0.00, 0.20)	0.229
Receiving health information through email	2.54 (1.05)	2.62 (1.08)	0.04 (−0.06, 0.14)	1.000
Receiving health information through text messaging	2.83 (0.99)	2.88 (1.06)	0.02 (−0.08, 0.11)	1.000
Receiving health information from social media	2.76 (1.05)	2.85 (1.05)	0.05 ( −0.04, 0.14)	1.000
Receiving health information from mobile app	2.82 (0.98)	2.84 (1.01)	−0.01 (−0.10, 0.08)	1.000

Personality types (introverts vs. extroverts) was considered as independent variables, with variable on each line taken as the dependent variable. After completing all the regressions, the Holm-Bonferroni method was used to adjust the *p* values from the regressions

^a^Adjusted for age, education, and perceived social rank

^**^Adjusted *p* values were *p*-values calculated by Holm–Bonferroni method

## Discussion

Conducted in a context where social distancing measures are still enforced, this study provides a thorough examination of the varied responses of introverts and extroverts to the pandemic, exploring aspects such as perceptions of its impact on lifestyle and health, psychological status, COVID-19 knowledge, e-health literacy, infection status, and future pandemic preparedness preferences. Uniquely employing both self-reported changes in mental health since the onset of the pandemic and cross-sectional assessment on psychological outcomes, our research uncovers that while introverts and extroverts share similar levels of changes in psychological strain from the pandemic, the roots and nature of their stress differ in ways not previously identified. Specifically, we found that introverts experience heightened anxiety, depression, and fear, whereas extroverts predominantly face a loss of control. Additionally, our findings offer novel insights into their differing perceptions of lifestyle and health impacts, with extroverts showing a greater increase in having meals at home and introverts experiencing a greater decline in sleep quality. Moreover, extroverts also demonstrated greater COVID-19 awareness and prevention knowledge. Furthermore, extroverts showed a stronger inclination towards digital solutions for future pandemic preparedness, including online consultations with doctors, instant personalized health advice from online chatbots, online courses, instant streaming courses, medicine delivery, online shopping, and food delivery, highlighting a distinct preference for technology-enhanced resilience strategies.

Our study analyzed the psychological impact of the pandemic, capturing both longitudinal self-reported changes and cross-sectional statuses. Notably, both introverts and extroverts experienced increased mental distress from COVID-19, with no significant difference in the extent of this increase. However, introverts reported more anxiety, depression, and fearfulness, which align with previous findings [8–11, 26], while extroverts felt a loss of control. The findings suggest both of them have increased mental burden, but the source of distress diverges. These differences align with their coping strategies under stressor; introverts lean towards avoidant methods [27, 28], which have been found associated with exacerbating levels of anxiety, depression, and fear [29, 30], whereas extroverts prefer direct strategies, leading to loss of control when ineffective [31, 32]. Thus, while the pandemic’s psychological burden is similar for both groups, the specific distress types vary significantly. This finding emphasizes the necessity for personalized mental health strategies for introverts and extroverts during pandemics. A recent study found that extraversion moderated the relationship between changes in social media communication and changes in perceived control over life [33], suggesting that targeted social interventions could help mitigate extraversion’s impact on individuals’ sense of control. Health practitioners should tailor their focus in personality assessments, concentrating on managing overstimulation for introverts and loss of control for extroverts. Policymakers and public health officials must design interventions and communications to meet these diverse psychological needs, ensuring that mental health resources are tailored and accessible for various personality groups. This could involve providing extroverts with techniques to mitigate loss of control through their preferred social channels and helping introverts reduce overstimulation to effectively alleviate their mental health burden.

Regarding the changes of lifestyle and health from the COVID-19 pandemic, extroverts experienced a more significant increase in having a meal at home than introverts, possibly as they enjoy socializing and being around people [34–36]. During the pandemic, traditional face-to-face social activities were restricted owing to social distancing measures [37], causing extroverts to eat at home. In line with recent findings that extroverts typically experience better sleep quality [38], we found that sleep quality during the pandemic was not significantly altered among extroverts, but it declined among introverts; During lockdowns, the prolonged presence of household members, who often work from home and have constant meetings and considering the small living spaces typical for Hong Kong, can significantly reduce personal space and quiet time. Having quiet time during the day is critical to rest for introverts, and even simple conversations with others can cause overstimulation [39, 40]. Consequently, this lack of quiet can be especially taxing for introverts, impairing their ability to unwind and, subsequently, their sleep quality. This finding calls for personalized health strategies: enhancing sleep quality and managing overstimulation for introverts, and for extroverts, not only facilitating safe social interactions but also promoting nutritional education as they adapt to more frequent home meals. Policymakers and health practitioners should integrate these personality-specific needs into public health directives and interventions, such as virtual social dining experiences for extroverts and quiet space guidelines for introverts.

Extroverts showed a higher level of self-rated knowledge than introverts on COVID-19 and its prevention in our community setting. This has also been reported among university students in Kenya [41]. However, no difference was observed between extroverts and introverts on the level of e-health literacy. As a result, extroverts' higher knowledge levels may be due to their social nature and easier access to knowledge from their friends and families [42]. Lower levels of knowledge about COVID-19 and its prevention has been found to induce fear [43, 44], anxiety [44, 45], and depression [45]. A recent study similarly found that extraversion is positively associated with better self-rated health during the COVID-19 pandemic [46]. Our findings suggest improving introverts’ confidence in COVID-19-related knowledge and its prevention might be an approach to alleviate their psychological distress. The difference in COVID-19 knowledge between extroverts and introverts calls for diversified communication strategies to ensure all personality types are well-informed. Healthcare providers should offer targeted support to boost introverts' knowledge confidence, potentially reducing their pandemic-related distress. Policymakers and public health campaigns must employ a mix of direct and peer-to-peer information dissemination methods, leveraging digital platforms to bridge the knowledge gap and cater to both extroverts' social nature and introverts' preference for less interaction.

Finally, extroverts rated the importance of all COVID-19 preparations higher than introverts did, with statistical significance reached in online consultations with doctors, instant personalized health by online chatbots, instant streaming courses, online courses, medicine delivery, online shopping, and food delivery. extraversion was a predictor of more positive attitudes toward online counseling [47], aligning with recent evidence that extraversion is positively associated with online communication and participation [48]. Online consultations require instantaneous responses; thus, introverts may find them too stressful and fast-paced [47]. This may explain why extroverts value online consultations more. Additionally, extroverts’ positive attitudes toward chatbots may be related to their higher degree of anthropomorphization and affection toward robots [49]. Consequently, introverts may place less importance on instant chat with chatbots. During the pandemic, extroverts were found to be more motivated to study, adaptable [50], and satisfied with online learning compared to introverts [51], who felt lonely. Our study supports this finding, as extroverts perceived higher importance in online courses and instant streaming. Additionally, extroverts are more likely to trust e-commerce than introverts and have a more positive attitude toward online shopping, medicine delivery, and food delivery [52, 53]. The distinction between extroverts and introverts underscores the importance of customizing health strategies and policies to meet their differing needs. Extroverts, with a preference for digital communication, are more inclined to engage with telehealth and online services if these platforms incorporate interactive features like social media and gamification. This approach can boost their engagement and readiness for health services. Conversely, introverts favor less intrusive services with minimal social interaction, benefiting from self-guided telehealth options, asynchronous communication with providers, and a strong emphasis on privacy and security. Future telehealth or digital platforms should incorporate interactive features customized to users' personality preferences, such as enabling personality type selection for tailored interaction levels. This personalization approach promotes inclusivity and enhances the efficacy of health strategies, improving pandemic preparedness and health management.

### Limitations and future directions

Two limitations of our study are worth noting. First, our approach to measuring personality was to use a single question focused on the extraversion dimension, which may seem simplistic and potentially inaccurate. However, we provided detailed descriptions of introverts and extroverts in the options, which could help mitigate response inaccuracies. Future studies are encouraged to employ more established instruments, such as the Big Five Inventory or the Myers-Briggs Type Indicator, to validate our findings. Additionally, research could explore other personality dimensions beyond extraversion or assess the reliability of using a single question to evaluate extraversion. Second, we could not include a larger sample of adults in Hong Kong owing to limited resources. Consequently, the generalizability of our findings may be limited, particularly in different cultural populations. Thus, including participants from multiple nations to identify global trends of personality differences in response to the pandemic and to make cross-country comparisons would be desirable. Additionally, future studies could develop individualized interventions based on our findings and analyze their effectiveness in addressing relative health concerns.

## Conclusions

In conclusion, our study highlights the significant impact of personality traits on people’s behaviors, mental well-being, and preferred preparations during a pandemic. To improve pandemic-related interventions, researchers should consider personality traits as a crucial factor in their development. Mental health professionals should also consider personality when developing treatment approaches that address the diverse needs of introverts and extroverts. Policymakers can use our findings to consider various methods of disseminating COVID-19 information and developing preparations to improve that consider the impact of personality traits on the well-being of both types. Additionally, our insights on preparation preferences for online consultations, health services, and interventions can guide the development of effective pandemic interventions and treatment approaches. Future studies should further investigate how personality affects the effectiveness of telehealth and e-health interventions to improve outcomes for introverts and extroverts. By incorporating these actionable snippets into pandemic response plans, governments and practitioners can develop more effective pandemic interventions and treatment approaches that consider the impact of personality traits on people’s behaviors and mental well-being during a pandemic.

## Data Availability

The datasets used and/or analysed during the current study are available from the corresponding author on reasonable request.

## Abbreviations

WHO: World Health Organization
BMI: Body mass index
